# Outcomes After Resection of Adenocarcinoma of the Gastric Cardia by Surgical Approach

**DOI:** 10.1245/s10434-025-17431-5

**Published:** 2025-05-08

**Authors:** Kelly R. Bates, Ryan C. Jacobs, Norah N. Zaza, Marjorie R. Liggett, Saieesh A. Rao, Dominic J. Vitello, David J. Bentrem

**Affiliations:** 1 Department of Surgery, Northwestern University Feinberg School of Medicine, Chicago, IL USA; 2 Department of Surgery, Jesse Brown Veterans Administration Medical Center, Chicago, IL USA

**Keywords:** Gastric adenocarcinoma, Total gastrectomy, Esophagectomy, Surgical approach, Stomach cancer

## Abstract

**Background:**

Total gastrectomy and esophagectomy are commonly used surgical approaches for cardia gastric adenocarcinoma (GA) resection. However, the preferred approach remains unclear. The objectives of this study were to identify predictors of receipt of surgical approach type and compare surgical approach outcomes.

**Patients and Methods:**

Patients with stage IB–IIIC cardia GA from 2004 to 2017 were identified within the National Cancer Database. Patients were compared on the basis of receipt of total gastrectomy versus partial gastrectomy with esophagectomy. Predictors of receiving esophagectomy were identified using multivariable logistic regression. Predictors associated with overall survival (OS) were assessed using a multivariable Cox proportional hazards model.

**Results:**

A total of 9841 patients were included. More patients underwent esophagectomy compared with total gastrectomy (77.2% vs. 22.8%). Surgical approach utilization did not vary significantly over time (*p* = 0.6). Patients who were non-white or female (OR 0.8, 95% CI 0.7–0.9) were less likely to receive esophagectomy. The median number of lymph nodes resected was greater for total gastrectomy versus esophagectomy (18 vs. 15, *p* < 0.01). There was no difference in resection margins (93.6% vs. 94.5%, *p* = 0.3) or 30-day mortality (3.0% vs. 2.5%, *p* = 0.2). Total gastrectomy and esophagectomy had similar OS (40.2 vs. 40.1 months, *p* = 0.7). On multivariate analysis, there was no difference in survival for total gastrectomy versus esophagectomy (HR 1.0, 95% CI 0.9–1.0).

**Conclusions:**

Utilization of total gastrectomy and esophagectomy has remained stable over time with esophagectomy being more utilized. These approaches exhibit similar oncologic outcomes for proximal GA. Surgeons should consider long-term outcomes, such as quality of life and nutritional status, when selecting an approach.

**Supplementary Information:**

The online version contains supplementary material available at 10.1245/s10434-025-17431-5.

Gastric adenocarcinoma (GA) is the fifth leading cause of cancer worldwide and is associated with high mortality.^1^ It is possible to categorize GA on the basis of its anatomic location, with cardia tumors situated at the esophagogastric junction and non-cardia tumors located more distally within the stomach. Though the incidence of non-cardia GA has been declining in the USA in recent years, the incidence of cardia GA has increased approximately sevenfold.^2,3^ Neoplasms in the gastric cardia are also associated with worse functional and long-term outcomes, with one study reporting a 5-year survival rate as low as 25% for patients with cardia GA.^4–6^ As a result of this increasing incidence and high mortality rate, there exists discourse regarding the most effective surgical approach for the treatment of cardia GA.

Total gastrectomy and partial gastrectomy with esophagectomy are two commonly used surgical approaches for resection of cardia GA. Total gastrectomy involves the resection of the entire stomach, whereas partial gastrectomy with esophagectomy resects only the portion of the stomach which is necessary. While total gastrectomy has been the historically more commonly utilized surgical approach, a potential benefit to performing esophagectomy is to preserve digestive function by keeping part of the stomach intact.^7^ As a result, partial gastrectomy with esophagectomy may minimize postoperative weight loss compared with total gastrectomy.^8^ Additionally, partial gastrectomy with esophagectomy may allow for more robust lymph node harvest by including mediastinal lymph nodes as part of the resection.^9^ Although these procedures are commonly performed, they are associated with serious mortality rates and postoperative complications, including nutritional deficiency, weight loss, and reflux esophagitis.^10–14^ Therefore, it is of great interest to identify a surgical approach that optimizes postoperative outcomes and quality of life for patients with cardia GA.

There is insufficient evidence to support one surgical approach over the other. Though various studies have sought to identify the most effective surgical approach for cardia GA resection, the literature shows varied results as to which approach is associated with preferable outcomes, therefore contributing to the uncertainty surrounding treatment for this type of neoplasm.^8,9,15–26^ The objectives of this study are to (1) identify predictors of receipt of total gastrectomy versus partial gastrectomy with esophagectomy, (2) compare oncologic quality measures by surgical approach, and (3) evaluate survival outcomes by surgical approach.

## Patients and Methods

### Study Design and Data Source

This study is a retrospective observational cohort analysis of patients diagnosed with clinical stage IB–IIIC gastric adenocarcinoma localized to the cardia from 2004 to 2017 using the 2020 National Cancer Database (NCDB) gastric participant use file (PUF). The NCDB is a national clinical oncology database sourced from hospital registry data collected in more than 1500 Commission on Cancer (CoC)-accredited facilities in the USA and Puerto Rico. Trained individuals extract information from patient records diagnosed with all types of cancer at participating facilities. In so doing, approximately 72% of all newly diagnosed cancers are captured within the database.^27,28^ Patients were categorized into those who underwent total gastrectomy with or without esophagectomy versus those who underwent partial or subtotal gastrectomy with esophagectomy. The Northwestern University Institutional Review Board deemed this study exempt from review as the data were deidentified.

### Participants

Patients with a histologically confirmed diagnosis of clinical stage IB–IIIC cardia GA with a known treatment strategy who underwent surgical resection with either total gastrectomy with or without esophagectomy, or partial or subtotal gastrectomy with esophagectomy, were included for analysis. Patients with less than clinical stage IB GA, metastatic GA, or unknown clinical stage were excluded (Supplementary Fig. 1). Clinical staging was determined using the American Joint Committee on Cancer 8th Edition Staging System.

### Surgical Approach

The aim of the study was to compare total gastrectomy with partial gastrectomy with esophagectomy. We used the code descriptions “gastrectomy, not otherwise specified (NOS) with removal of a portion of esophagus” and “partial and subtotal gastrectomy with removal of a portion of esophagus” to categorize the esophagectomy group, and we used the variables “near-total or total gastrectomy, NOS,” “near-total gastrectomy,” “total gastrectomy,” and “near-total or total gastrectomy with removal of a portion of esophagus” to categorize the total gastrectomy group (Supplementary Table 1). Total gastrectomy with esophagectomy was presumed to not represent esophagectomy because the entire stomach was resected in these patients, therefore more closely representing the more invasive total gastrectomy group.

### Patient Demographic and Clinical Characteristic Variables

Patient demographic and clinical characteristic variables of interest included “sex,” “race/ethnicity,” “primary insurance type,” “age quartile,” “income quartile,” “Charlson–Deyo score,” “prognostic stage group,” “receipt of adjuvant therapy,” “receipt of neoadjuvant therapy,” “urban/rural,” and “facility.” Charlson–Deyo score was reported as an ordinal variable. The prognostic stage group, receipt of adjuvant therapy, receipt of neoadjuvant therapy, urban/rural, and facility variables were primarily derived.

### Outcomes

The primary outcome of interest was surgical approach. The secondary outcome of interest was overall survival (OS), as defined by a time-to-event variable using “last vital status” as the binary outcome variable and “last contact, months from diagnosis” as the time variable. Oncologic quality measures included the categorical variable “resection margins”; the binary outcome variables “received adjuvant therapy per National Comprehensive Cancer Network (NCCN) recommendation,” “30-day mortality,” “90-day mortality,” and “guidance-concordant lymph node harvest”; and the continuous variables “lymph nodes examined,” “travel distance,” “time to treatment,” “time to adjuvant therapy after surgery,” and “length of stay.”

### Statistical Analysis

Descriptive statistics were calculated for the final cohort of patients. Bivariate comparisons for continuous variables were assessed using *t*-tests, and categorical variables were assessed with chi-squared tests. A multivariable logistic regression model was estimated to determine predictors of receipt of partial gastrectomy with esophagectomy compared with total gastrectomy. Variables were included in the model on the basis of clinical- and literature-reported relevance.^24,29^ These variables consisted of race, age, sex assigned at birth, income quartile, insurance status, clinical stage, rural or urban community, and facility type. Odds ratios (ORs) were calculated for each of these variables. Rates of utilization of surgical approach by year were compared using a Cochran–Armitage test for trend. The Kaplan–Meier method was used to estimate OS, and differences in OS were assessed using the log-rank test. A multivariable Cox proportional hazards model was utilized to determine predictors associated with OS, and the parameter estimates were in the form of hazards ratios (HRs) for included covariates. A sensitivity analysis comparing rates of guidance-concordant lymph node harvest was conducted using a multivariable logistic regression model, with ORs calculated for each covariate. For each of the models, 95% confidence intervals (CIs) and *p* values were calculated for all ORs and HRs. A *p* value of less than 0.05 was considered statistically significant. Patients with incomplete or missing covariates were dropped from the regression analysis. All analyses were conducted using Stata SE 16.1 (College Station, Texas).

## Results

### Patient Demographic and Clinical Characteristics

The final cohort for analysis following inclusion and exclusion criteria included 9841 patients from 1041 hospitals. The patients were predominantly male (82.5%), white (86.4%), and treated at academic centers (51.3%). The majority of patients had Charlson–Deyo scores of 0 (69.0%) and were in the stage III prognostic group (69.0%). Most patients received partial gastrectomy with esophagectomy (77.2%), while 22.8% of patients received total gastrectomy. Notably, the partial gastrectomy with esophagectomy cohort had a greater proportion of male (83.5% vs. 79.02%) and non-Hispanic white (88.7% vs. 78.7%) patients compared with the total gastrectomy group. The total gastrectomy cohort had a slightly greater proportion of patients with Charlson–Deyo 0 (70.4% vs. 68.6%) and patients who received treatment at low-volume nonacademic facilities (45.0% vs. 42.7%) compared with the partial gastrectomy with esophagectomy cohort (Table 1).

**Table 1:** Demographics and clinical characteristics of patients undergoing surgical resection for clinical IB–IIIC cardia gastric adenocarcinoma by surgical approach

Demographic	Surgical approach		Total
	Total gastrectomy *N* (%)	Partial gastrectomy with esophagectomy *N* (%)	
Total	2245 (22.81)	7596 (77.19)	9841 (100.00)
*Age (years)*			
19–57	578 (25.75)	1819 (23.95)	2397 (24.36)
58–64	491 (21.87)	1831 (24.10)	2322 (23.60)
65–71	550 (24.50)	2037 (26.82)	2587 (26.29)
72–90	626 (27.88)	1909 (25.13)	2535 (25.76)
*Sex*			
Male	1774 (79.02)	6342 (83.49)	8116 (82.47)
Female	471 (20.98)	1254 (16.51)	1725 (17.53)
*Race/ethnicity*			
White	1767 (78.71)	6737 (88.69)	8504 (86.41)
Black	124 (5.52)	187 (2.46)	311 (3.16)
Hispanic	120 (5.35)	208 (2.74)	328 (3.33)
Asian	99 (4.41)	112 (1.47)	211 (2.14)
Other	135 (6.01)	352 (4.63)	487 (4.95)
*Charlson–Deyo*			
0	1581 (70.42)	5212 (68.62)	6793 (69.03)
1	487 (21.69)	1748 (23.01)	2235 (22.71)
2	177 (7.88)	636 (8.37)	813 (8.26)
*Primary insurance type*			
Uninsured	47 (2.13)	131 (1.74)	178 (1.83)
Private	1025 (46.40)	3477 (46.23)	4502 (46.27)
Medicaid	119 (5.39)	385 (5.12)	504 (5.18)
Medicare	990 (44.82)	3429 (45.59)	4419 (45.42)
Other Government	28 (1.27)	99 (1.32)	127 (1.31)
*Income (USD)*			
< $40,227	294 (15.04)	871 (13.52)	1165 (13.87)
$40,227–50,353	406 (20.77)	1394 (21.64)	1800 (31.43)
$50,354–60,332	454 (23.22)	1619 (25.13)	2073 (24.68)
$60,333+	801 (40.97)	2559 (39.72)	3360 (40.01)
*Stage*			
I	294 (13.10)	1168 (15.38)	1462 (14.86)
II	911 (40.58)	2836 (37.34)	3747 (38.08)
III	1040 (46.33)	3592 (47.29)	4632 (47.07)
*Urban/rural*			
Rural	124 (5.80)	431 (6.03)	555 (5.98)
Urban	2013 (94.20)	6717 (93.97)	8730 (94.02)
*Facility*			
High-volume academic	820 (43.71)	2946 (44.70)	3766 (44.48)
High-volume nonacademic	212 (11.30)	829 (12.58)	1041 (12.30)
Low-volume nonacademic	844 (44.99)	2815 (42.72)	3659 (43.22)
*Receipt of neoadjuvant therapy*			
Chemotherapy	580 (25.84)	890 (11.72)	1470 (14.94)
Chemoradiotherapy	930 (41.43)	4205 (55.36)	5135 (52.18)
No	735 (32.74)	2501 (32.93)	3236 (32.88)
Receipt of adjuvant therapy			
Yes	574 (25.57)	1474 (19.40)	2048 (20.81)
No	1671 (74.43)	6122 (80.60)	7793 (79.19)

*IQR* interquartile range, *N* number, *NCCN* National Comprehensive Cancer Network, *OS* overall survival

### Unadjusted Quality Measure Outcomes

A greater percentage of patients in the total gastrectomy group had at least 16 resected lymph nodes compared with patients in the partial gastrectomy with esophagectomy group (57.7% vs. 47.9%, *p* < 0.01). The median number of lymph nodes resected was also greater for the total gastrectomy group compared with those who received partial gastrectomy with esophagectomy (18 vs. 15, *p* < 0.01). Resection margins, including R0 margins, were similar for both the total gastrectomy and the partial gastrectomy with esophagectomy groups (93.6% vs. 94.5%, *p* = 0.3). All other quality measures, including time to treatment, time to adjuvant therapy after surgery, travel distance, receipt of adjuvant therapy per NCCN recommendation, 30- and 90-day mortality, and length of stay, were not statistically significantly different (Table 2).

**Table 2:** Unadjusted Commission on Cancer (CoC) quality of care measures of patients undergoing surgical resection for clinical IB–IIIC cardia gastric adenocarcinoma by surgical approach

Quality measures	Surgical approach		Total	*p* value
	Total gastrectomy	Partial gastrectomy with esophagectomy		
Resection margins, *N* (%)				
R0	1973 (93.64)	6872 (94.45)	8845 (94.27)	0.29
R1	126 (5.98)	386 (5.31)	512 (5.46)	
R2	8 (0.38)	18 (0.25)	26 (0.28)	
Received adjuvant therapy per NCCN recommendation, *N* (%)				
Yes	208 (13.91)	586 (12.50)	794 (12.84)	0.15
No	1287 (86.09)	4103 (87.50)	5390 (87.16)	
30-day mortality, *N* (%)				
Alive	2165 (97.00)	7376 (97.55)	9541 (97.43)	0.15
Died	67 (3.00)	185 (2.45)	252 (2.57)	
90-day mortality, *N* (%)				
Alive	2081 (93.49)	7046 (93.55)	9127 (93.53)	0.92
Died	145 (6.51)	486 (6.45)	631 (6.47)	
Guidance-concordant lymph node harvest, *N* (%)				
< 16	949 (42.27)	3958 (52.11)	4907 (49.86)	< 0.01
≥ 16	1296 (57.73)	3638 (47.89)	4934 (50.14)	
Lymph nodes examined, median (IQR)	18 (11–25)	15 (9-22)	16 (10-22)	< 0.01
Travel distance (miles), median (IQR)	16.1 (6.6–41.9)	17.55 (7.3–44.9)	17.2 (7.1–44.1)	0.81
Time to treatment (weeks), median (IQR)	4.86 (3.14–6.86)	5.00 (3.43–6.86)	4.86 (3.43–6.86)	0.94
Time to adjuvant therapy after surgery (days), median (IQR)	86 (59–115)	91 (66–123)	90 (63–122)	0.23
Length of stay (days), median (IQR)	9 (7–13)	9 (7–14)	9 (7–13)	0.30

*IQR* interquartile range, *N* number, *NCCN* National Comprehensive Cancer Network, *OS* overall survival

### Lymph Node Harvest Sensitivity Analysis

When adjusting for neoadjuvant treatment type and other covariates, partial gastrectomy with esophagectomy was statistically significantly associated with removal of fewer than 16 lymph nodes (OR 0.8, 95% CI 0.7–0.8). Receipt of neoadjuvant chemotherapy was associated with resection of 16 or more lymph nodes when compared with no neoadjuvant therapy (OR 1.5, 95% CI 1.3–1.8), whereas receipt of neoadjuvant chemoradiotherapy was associated with resection of less than 16 nodes (OR 0.8, 95% CI 0.7–0.9) (Supplementary Table 2).

### Predictors and Rates of Receipt of Surgical Approach

On multivariable logistic regression, patients who were less likely to receive partial gastrectomy with esophagectomy identified as a non-white race or ethnicity, such as non-Hispanic Black (OR 0.4, 95% CI 0.3–0.6), Asian (OR 0.3, 95% CI 0.2–0.5), Hispanic (OR 0.4, 95% CI 0.3–0.5), and other races or ethnicities (OR 0.7, 95% CI 0.5–0.8). Likewise, patients who were female compared with male (OR 0.8, 95% CI 0.7–0.9) were also less likely to receive partial gastrectomy with esophagectomy (Table 3). On Cochran–Armitage test for trend, rates of utilization of total gastrectomy versus partial gastrectomy with esophagectomy did not vary significantly over time (*p* = 0.6) (Table 4).

**Table 3:** Multivariable logistic regression of predictors of receipt of partial gastrectomy with esophagectomy compared with total gastrectomy

	Odds ratio		95% confidence interval	*p* value
*Race*				
Non-Hispanic white	1	Reference		
Non-Hispanic Black	0.43		0.32–0.58	< 0.01
Hispanic	0.40		0.30–0.53	< 0.01
Asian	0.32		0.22–0.46	< 0.01
Other	0.66		0.52–0.83	< 0.01
*Income*				
< $40,227	1	Reference		
$40,227–50,353	1.07		0.88–1.31	0.50
$50,354–60,332	1.07		0.88–1.31	0.49
$60,333+	1.00		0.83–1.21	0.99
*Insurance*				
Private	1	Reference		
Uninsured	0.81		0.53–1.22	0.31
Medicaid	1.01		0.77–1.34	0.92
Medicare	1.04		0.89–1.21	0.67
Other government	0.99		0.59–1.67	0.97
Charlson–Deyo score	1.08		0.99–1.19	0.09
Age	1.00		0.99–1.01	0.55
*Sex*				
Male	1	Reference		
Female	0.78		0.67–0.90	< 0.01
Stage				
I/II	1	Reference		
III	1.04		0.93–1.17	0.49
*Rural/urban*				
Rural	1	Reference		
Urban	1.09		0.85–1.40	0.48
*Facility*				
High-volume academic	1	Reference		
High-volume nonacademic	1.15		0.94–1.39	0.17
Low-volume nonacademic	0.98		0.87–1.11	0.77

*IQR* interquartile range, *N* number, *NCCN* National Comprehensive Cancer Network, *OS* overall survival

**Table 4:** Rates of surgical approaches over time; Cochran–Armitage test for trend of rates of surgical approach, by year

Year	Surgical approach		Total	*p* value
	Total gastrectomy	Partial gastrectomy with esophagectomy		
2004, *N* (%)	56 (20.29)	220 (79.71)	276 (100.00)	0.56
2005, *N* (%)	68 (23.94)	216 (76.06)	284 (100.00)	
2006, *N* (%)	71 (20.46)	276 (79.54)	347 (100.00)	
2007, *N* (%)	86 (23.24)	284 (76.76)	370 (100.00)	
2008, *N* (%)	110 (25.00)	330 (75.00)	440 (100.00)	
2009, *N* (%)	153 (24.64)	468 (75.36)	621 (100.00)	
2010, *N* (%)	185 (23.36)	607 (76.64)	792 (100.00)	
2011, *N* (%)	181 (21.47)	662 (78.53)	843 (100.00)	
2012, *N* (%)	185 (21.31)	683 (78.69)	868 (100.00)	
2013, *N* (%)	207 (20.91)	783 (79.09)	990 (100.00)	
2014, *N* (%)	238 (23.63)	769 (76.37)	1007 (100.00)	
2015, *N* (%)	252 (24.16)	791 (75.84)	1043 (100.00)	
2016, *N* (%)	218 (21.48)	797 (78.52)	1015 (100.00)	
2017, *N* (%)	235 (24.87)	710 (75.13)	945 (100.00)	

*IQR* interquartile range, *N* number, *NCCN* National Comprehensive Cancer Network, *OS* overall survival

### Survival Analysis

**Fig. 1:**
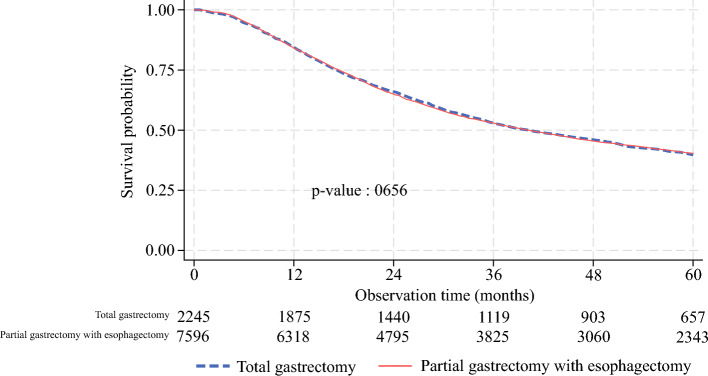
Kaplan–Meier survival curve estimates for patients receiving total gastrectomy and partial gastrectomy with esophagectomy

The total gastrectomy and the partial gastrectomy with esophagectomy groups had similar OS (40.2 vs. 40.1 months, *p* = 0.7) and length of follow-up (35.9 vs. 36.3 months) (Table 6), as depicted by the Kaplan–Meier survival curve estimates (Fig. 1). On multivariable Cox regression, for patients receiving partial gastrectomy with esophagectomy, the associated OS was not significantly different than that for patients receiving total gastrectomy (HR 1.0, 95% CI 0.9–1.0). Predictors of lower OS included higher Charlson–Deyo scores (HR 1.1, 95% CI 1.1–1.2) compared with Charlson–Deyo 0, prognostic stage III (HR 1.4, 95% CI 1.3–1.5) cancer at diagnosis compared with prognostic stage I/II, and treatment at low-volume nonacademic (HR 1.2, 95% CI 1.1–1.3) or high-volume nonacademic (HR 1.3, 95% CI 1.1–1.4) facilities compared to treatment at high-volume academic facilities (Table 5).

**Table 5:** Multivariable Cox proportional hazards regression of patients undergoing surgical resection for clinical IB–IIIC cardia gastric adenocarcinoma, by surgical approach

	Hazard ratio		95% confidence interval	*p* value
*Surgical approach*				
Total gastrectomy	1	Reference		
Partial gastrectomy with esophagectomy	0.96		0.89–1.03	0.28
*Race*				
Non-Hispanic white	1	Reference		
Non-Hispanic Black	0.81		0.68–0.98	0.03
Hispanic	0.83		0.69–1.00	0.06
Asian	0.88		0.68–1.13	0.31
Other	1.02		0.90–1.17	0.72
*Income*				
< $40,227	1	Reference		
$40,227–50,353	1.00		0.91–1.11	0.97
$50,354–60,332	0.97		0.88–1.07	0.57
$60,333+	0.85		0.78–0.94	< 0.01
*Insurance*				
Private	1	Reference		
Uninsured	1.22		0.98–1.52	0.08
Medicaid	1.09		0.93–1.27	0.28
Medicare	1.05		0.97–1.14	0.21
Other government	1.03		0.78–1.37	0.82
Charlson–Deyo score	1.11		1.06–1.16	< 0.01
Age	1.02		1.01–1.02	< 0.01
*Sex*				
Male	1	Reference		
Female	0.88		0.81–0.95	< 0.01
*Prognostic stage*				
I/II	1	Reference		
III	1.37		1.29–1.46	< 0.01
*Rural/urban*				
Rural	1	Reference		
Urban	0.93		0.82-1.05	0.24
*Facility*				
High-volume academic	1	Reference		
High-volume nonacademic	1.25		1.13–1.37	< 0.01
Low-volume nonacademic	1.20		1.13–1.28	< 0.01
*Receipt of neoadjuvant therapy*				
No	1	Reference		
Chemotherapy	0.86		0.78–0.95	< 0.01
Chemoradiotherapy	0.87		0.81–0.95	< 0.01
*Receipt of adjuvant therapy*				
No	1	Reference		
Yes	0.98		0.90–1.07	0.71

*IQR* interquartile range, *N* number, *NCCN* National Comprehensive Cancer Network, *OS* overall survival

## Discussion

The results of this study show that both total gastrectomy and partial gastrectomy with esophagectomy demonstrate clinically similar survival outcomes for treatment of cardia GA. The quality of oncologic resection was also similar between the comparison groups. Though these surgical approaches are equivalent in oncologic outcome, it is important to note that they may differ in regard to functional outcomes (Table 6).

**Table 6:** Summary of unadjusted survival outcomes of patients undergoing surgical resection for clinical IB–IIIC cardia gastric adenocarcinoma, by surgical approach

	Surgical approach		
	Cohort	Total gastrectomy	Partial gastrectomy with esophagectomy
Length of follow-up, median (IQR)	36.24 (16.56–68.24)	35.88 (16.33–65.71)	36.34 (16.62–68.96)
OS, median (IQR)	40.08 (17.35–135.69)	40.15 (17.08–133.55)	40.08 (17.41–136.31)

*IQR* interquartile range, *N* number, *NCCN* National Comprehensive Cancer Network, *OS* overall survival

These surgical approaches uphold comparable oncologic quality, according to the Commission on Cancer (CoC) Quality of Care Measures. The CoC has published the Quality of Care Measures to ensure that accredited hospitals maintain a high standard of care for cancer patients, with certain quality assurance criteria applying to each type of cancer. According to NCCN guidelines for gastric cancer resection, it is expected that at least 16 lymph nodes are resected and examined pathologically, though it is ideal to resect 30 nodes. Though there are some statistically significant differences regarding the CoC Quality of Care Measures, particularly with the total gastrectomy group having more resected lymph nodes, both surgical approaches appear to demonstrate similar oncologic quality.

Though there are some studies that argue for the superiority of one surgical approach over the other, this study supports the majority of the literature, which implies that there is no significant difference in the survival outcomes for patients receiving total gastrectomy compared with partial gastrectomy with esophagectomy. Though some systematic reviews indicate that there may be a quality-of-life benefit for total gastrectomy compared with partial gastrectomy with esophagectomy, these studies also reveal that the oncologic benefit for total gastrectomy appears to be limited.^21,22^ Likewise, one meta-analysis suggests that total gastrectomy is associated with improved 30-day mortality and overall survival compared with partial gastrectomy with esophagectomy. However, following the exclusion of two large studies, these results were no longer statistically significant, suggesting that there may be bias in the studies used in the meta-analysis.^18^ Furthermore, improvements in postoperative complication management after esophagectomy have likely ameliorated this discrepancy. Overall, the results from this study are in accordance with most studies in the literature, which state that survival and postoperative outcomes are comparable for both total gastrectomy and partial gastrectomy with esophagectomy.^9,17,19,20,23–25^

Though this study demonstrates that OS following total gastrectomy or partial gastrectomy with esophagectomy is similar, it is important to note that there are differences regarding the predictors of receiving these surgical approaches. For patients with certain demographic backgrounds (insurance status and socioeconomic status), these results may be indicative of a disparity in regard to accessing certain treatment types. Furthermore, this study highlights the impact of social determinants of health on postoperative outcomes, especially since patients treated at nonacademic facilities were more likely to have shorter OS, as demonstrated by the Cox proportional hazards model.

Given the location of cardia GA, surgical resection of these tumors poses a considerable concern regarding postoperative nutrition. In the case of total gastrectomy, removing the stomach not only interferes with the chemical and mechanical breakdown of food, but it also drastically decreases the amount of circulating ghrelin, which is an appetite-stimulating hormone that is 70% excreted by the stomach.^30^ As a result, some surgeons may prefer the alternative surgical approach of resecting part of the stomach and part of the esophagus to reconstruct the gastric tract and preserve some stomach function. Notably, Tsumura et al. argues that laparoscopy-assisted proximal gastrectomy with esophagogastrostomy by the double-flap technique is preferable to laparoscopy-assisted total gastrectomy in terms of postoperative nutritional maintenance.^8^ Likewise, the Sentinel Node Oriented Tailored Approach randomized clinical trial demonstrated that patients who received stomach-preserving surgery had improved quality of life and postoperative nutrition compared with patients who received standard gastrectomy.^31^ Given the comparable postoperative survival outcomes for total gastrectomy and partial gastrectomy with esophagectomy, surgeons may consider these nutritional concerns, along with patients’ personal goals and quality of life, when selecting a surgical approach.

It is important to consider that there are additional factors and treatment modalities that contribute to patients’ postoperative outcomes and OS. For instance, neoadjuvant treatment, in addition to surgical resection, has been shown to be associated with improved OS for patients with cardia GA.^32^ Likewise, reconstruction method following resection may have considerable impacts on postoperative morbidity, functional outcomes, or quality of life. For instance, one concluded phase III randomized controlled trial has sought to assess the postoperative impact of utilizing a Roux-en-Y pouch reconstruction compared with a conventional Roux-en-Y following resection of gastric cancer.^33^ Similarly, a recent meta-analysis comparing Roux-en-Y with and without a jejunal J-pouch reconstruction following total gastrectomy has determined that Roux-en-Y with a J-pouch reconstruction may be associated with improved postoperative outcomes, such as decreased rates of dumping syndrome and heartburn.^34^ Additional studies have also investigated the impact of alternative reconstruction methods on quality-of-life outcomes. For instance, compared with the conventional Roux-en-Y, the double-tract reconstruction method may present with improved postoperative outcomes, such as time until starting a light diet.^35^ Therefore, our study demonstrates that surgical approach is only one of multiple contributing factors to postoperative survival, recovery, and remission.

One motivating factor in choosing one surgical approach over the other could be the quantity of lymph nodes obtained for pathologic assessment, which may be impacted by the type of neoadjuvant therapy utilized. To assess this, we performed a sensitivity analysis to compare the rates of guideline-concordant lymph node harvest (> 16 nodes). In the sensitivity analysis, it was shown that patients who received total gastrectomy more frequently had 16 or more lymph nodes pathologically examined compared with those who underwent partial gastrectomy with esophagectomy (57.7% vs. 47.9%, *p* < 0.01) (Table 2). It was hypothesized that this was due to differences in administration of neoadjuvant chemotherapy and chemoradiotherapy between the two surgical approaches. Upon analysis, the type of neoadjuvant therapy administered was associated with the quantity of lymph nodes retrieved, with neoadjuvant chemotherapy being predictive of resection of 16 or more nodes and neoadjuvant chemoradiotherapy being predictive of resection of less than 16 nodes. In the sensitivity analysis, we also found that total gastrectomy was associated with retrieval of more than 16 nodes. While partial gastrectomy with esophagectomy was associated with less than 16 nodes, patients who received partial gastrectomy with esophagectomy more frequently received neoadjuvant chemoradiotherapy, potentially confounding the association of operative approach with degree of nodal harvest. While overall survival was similar between the two groups in our study, other studies have shown greater nodal harvest to be associated with longer OS, which has been incorporated into NCCN guidelines.^36,37^ However, these studies have examined older cohorts and have not directly incorporated operative approach into their study designs. In all, nodal harvest may be a consideration when selecting an operative approach. However, further studies examining the influence of preoperative therapy and surgical approach on nodal harvest, and the effect of all these factors on survival in contemporary cohorts, may prove to be informative.

There are several limitations associated with this study. For instance, one limitation is the validity of the codes used for classifying procedure types. As detailed in the NCDB PUF data dictionary, there is a subset of codes that categorize various gastrectomy types with the resection of other organs. However, it is noted that the esophagus may or may not be included as an additionally resected organ in these codes. As a result, it is impossible to identify these patients within the database to ensure proper inclusion in the partial gastrectomy with esophagectomy comparison group. Additionally, there were individuals belonging to this subset of records who received a near total or total gastrectomy, but since the resection of unknown organs could potentially confound survival results, these patients were also excluded from the analysis. However, given the already large sample size in this study, the exclusion of these patients from the analysis likely had minimal effect on the results. Likewise, patients who received proximal gastrectomy were excluded from this analysis. An advantage of undergoing proximal gastrectomy would be to avoid the complications of esophageal anastomosis in the chest while also preserving the rest of the healthy stomach. However, given the lower utilization of this surgical approach, we chose to focus the analysis on the two most prevalent surgical approaches. In addition, since data from the NCDB PUF are only collected from participating hospitals, there is sampling bias associated with which hospitals and patient populations are included in analysis, which may underscore disparities observed in outcomes with marginalized groups as these groups are often under-represented in the NCDB. Finally, it is important to note that the NCDB lacks information pertaining to reconstruction methods, which affects structural outcomes, as well as postoperative morbidity, long-term outcomes, and patient-specific factors, which may be important to consider when selecting a surgical approach. However, morbidity is often reflected in survival outcomes, which are demonstrated to be similar in this study. Furthermore, advances in interventional techniques may attenuate the difficulties in managing previously challenging complications of gastrectomy with esophagectomy.

## Conclusions

While there are some patient-level predictors of utilization of total gastrectomy versus partial gastrectomy with esophagectomy, surgical oncologic quality measures and survival outcomes were similar between both surgical approaches. Given these results, both procedures serve as comparable surgical approaches for patients with cardia GA. However, additional factors, including nutritional status, health-related quality of life outcomes, and the surgeon’s personal experience, need to be further studied and considered to determine which surgical approach to utilize.

## Supplementary Information

Below is the link to the electronic supplementary material.

Supplementary file1 (DOCX 134 KB)
