# Supplementary material for: Outcomes After Resection of Adenocarcinoma of the Gastric Cardia by Surgical Approach
Source: Ann Surg Oncol. 2025 May 8;32(9):6783–93. doi: 10.1245/s10434-025-17431-5 (PMC12317889; doi:10.1245/s10434-025-17431-5)

**Supplemental Tables**

Table Legends

Supplemental Table 1: NCDB gastrectomy code descriptions included and excluded in the analysis

Supplemental Table 2: Multivariable logistic regression of predictors of resection of 16 or more lymph nodes compared to resection of less than 16 lymph nodes

Abbreviations appearing in tables

GA: Gastric adenocarcinoma

N: Number

NCDB: National Cancer Database

NOS: Not otherwise specified

Supplemental Table 1: NCDB gastrectomy code descriptions

| **NCDB Code** | **NCDB Code Description** | **Comparison Group** | **Total, N (% of patients with non-metastatic cardia GA)** | **Total, N (% of final cohort)** |
| --- | --- | --- | --- | --- |
| 40 | Near-total or total gastrectomy, NOS | Total gastrectomy | 203 (0.79) | 2,245 (22.81) |
| 41 | Near-total gastrectomy | Total gastrectomy | 38 (0.15) |  |
| 42 | Total gastrectomy | Total gastrectomy | 734 (2.86) |  |
| 52 | Near total or total gastrectomy with removal of a portion of esophagus | Total gastrectomy | 1,270 (4.95) |  |
| 50 | Gastrectomy, NOS with removal of a portion of esophagus | Partial gastrectomy with esophagectomy | 2,706 (10.55) | 7,596 (77.19) |
| 51 | Partial or subtotal gastrectomy with removal of a portion of esophagus | Partial gastrectomy with esophagectomy | 4,890 (19.07) |  |
| 00 | None; no surgery of primary site | Excluded | 10,657 (41.56) | 0 (0.00) |
| 10 | Local tumor destruction, NOS | Excluded | 6 (0.02) |  |
| 11 | Photodynamic therapy | Excluded | 12 (0.05) |  |
| 12 | Electrocautery; fulguration (includes use of hot forceps for tumor destruction) | Excluded | 5 (0.02) |  |
| 13 | Cryosurgery | Excluded | 11 (0.04) |  |
| 14 | Laser | Excluded | 8 (0.03) |  |
| 20 | Local tumor excision, NOS | Excluded | 134 (0.52) |  |
| 26 | Polypectomy | Excluded | 41 (0.16) |  |
| 27 | Excisional biopsy | Excluded | 225 (0.88) |  |
| 21 | Any combination of 20 or 26-27 with photodynamic therapy | Excluded | 4 (0.02) |  |
| 22 | Any combination of 20 or 26-27 with electrocautery | Excluded | 15 (0.06) |  |
| 23 | Any combination of 20 or 26-27 with cryosurgery | Excluded | 9 (0.04) |  |
| 24 | Any combination of 20 or 26-27 with laser ablation | Excluded | 11 (0.04) |  |
| 25 | Laser excision | Excluded | 5 (0.02) |  |
| 30 | Gastrectomy, NOS (partial, subtotal, hemi-) | Excluded | 1,120 (4.37) |  |
| 31 | Antrectomy, lower (distal-less than 40% of stomach) | Excluded | 37 (0.14) |  |
| 32 | Lower (distal) gastrectomy (partial, subtotal, hemi-) | Excluded | 615 (2.40) |  |
| 33 | Upper (proximal) gastrectomy (partial, subtotal, hemi-) | Excluded | 1,038 (4.05) |  |
| 60 | Gastrectomy with a resection in continuity with the resection of other organs, NOS | Excluded | 242 (0.94) |  |
| 61 | Partial or subtotal gastrectomy, in continuity with the resection of other organs | Excluded | 541 (2.11) |  |
| 62 | Near total or total gastrectomy, in continuity with the resection of other organs | Excluded | 293 (1.14) |  |
| 63 | Radical gastrectomy, in continuity with the resection of other organs | Excluded | 129 (0.50) |  |
| 80 | Gastrectomy, NOS | Excluded | 137 (0.53) |  |
| 90 | Surgery, NOS | Excluded | 437 (1.70) |  |
| 99 | Unknown if surgery performed | Excluded | 71 (0.28) |  |

Supplemental Table 2: Predictors associated with resection of 16 or more lymph nodes

|  | Odds Ratio |  | 95% Confidence Interval | p-value |
| --- | --- | --- | --- | --- |
| Procedure |  |  |  |  |
| Total gastrectomy | 1 | reference |  |  |
| Partial gastrectomy with esophagectomy | 0.75 |  | 0.66-0.84 | <0.01 |
| Neoadjuvant Therapy |  |  |  |  |
| None | 1 | reference |  |  |
| Chemotherapy only | 1.48 |  | 1.26-1.75 | <0.01 |
| Chemoradiotherapy | 0.79 |  | 0.71-0.88 | <0.01 |
| Race |  |  |  |  |
| Non-Hispanic white | 1 | reference |  |  |
| Non-Hispanic black | 1.26 |  | 0.93-1.71 | 0.13 |
| Hispanic | 1.44 |  | 1.06-1.95 | 0.02 |
| Asian | 1.53 |  | 1.04-2.26 | 0.03 |
| Other | 0.80 |  | 0.64-1.01 | 0.06 |
| Income |  |  |  |  |
| <$40,227 | 1 | reference |  |  |
| $40,227-$50,353 | 1.11 |  | 0.93-1.32 | 0.24 |
| $50,354-$60,332 | 1.16 |  | 0.97-1.37 | 0.10 |
| $60,333+ | 1.22 |  | 1.04-1.43 | 0.02 |
| Insurance |  |  |  |  |
| Private | 1 | reference |  |  |
| Uninsured | 0.78 |  | 0.53-1.14 | 0.20 |
| Medicaid | 1.16 |  | 0.91-1.48 | 0.24 |
| Medicare | 1.03 |  | 0.90-1.18 | 0.65 |
| Other Government | 0.98 |  | 0.63-1.54 | 0.93 |
| Charlson Deyo Score | 0.95 |  | 0.88-1.03 | 0.20 |
| Age | 1.00 |  | 0.99-1.00 | 0.41 |
| Sex |  |  |  |  |
| Male | 1 | reference |  |  |
| Female | 0.99 |  | 0.87-1.12 | 0.84 |
| Prognostic Stage |  |  |  |  |
| I/II | 1 | reference |  |  |
| III | 1.09 |  | 0.99-1.21 | 0.09 |
| Rural/Urban |  |  |  |  |
| Rural | 1 | reference |  |  |
| Urban | 0.95 |  | 0.76-1.17 | 0.62 |
| Facility |  |  |  |  |
| High volume academic | 1 | reference |  |  |
| High volume non-academic | 0.70 |  | 0.60-0.82 | <0.01 |
| Low volume non-academic | 0.46 |  | 0.42-0.51 | <0.01 |

**Supplemental Figure**

Figure Legends

Supplemental Figure 1: Flow diagram of cohort formation of patients undergoing surgical resection for clinical IB-IIIC cardia gastric adenocarcinoma

Abbreviations appearing in figures

GA: Gastric adenocarcinoma

NCDB: National Cancer Database

PUF: Participant user file

Supplemental Figure 1: Flow diagram of cohort formation


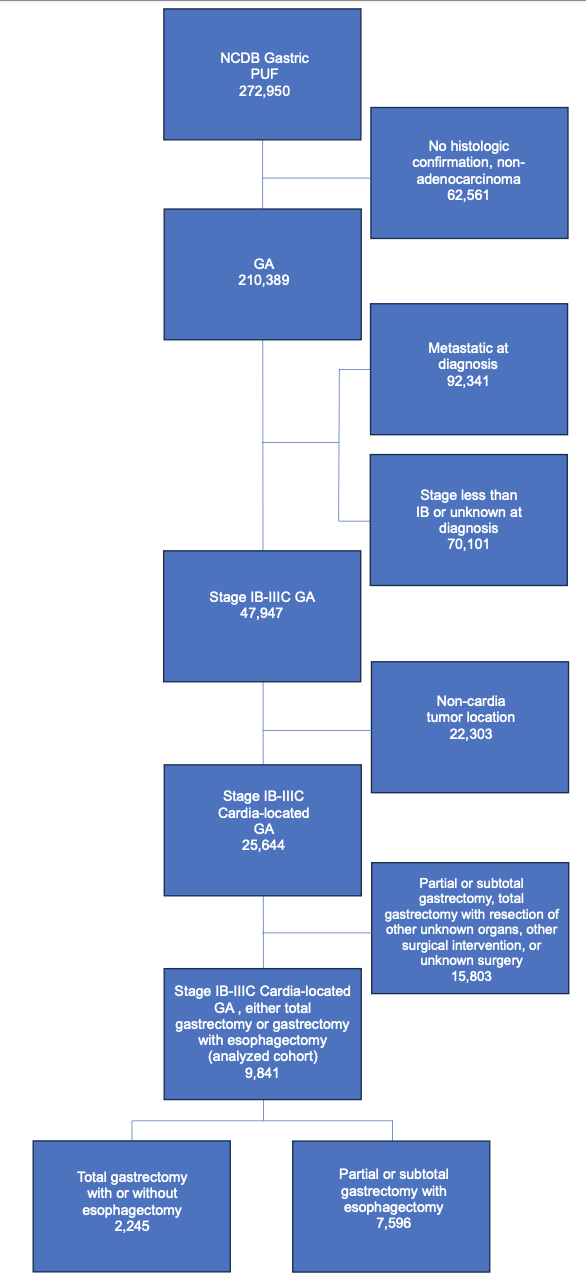

Supplement: Supplementary file 1 — Supplementary file1 (DOCX 134 KB) [file 10434_2025_17431_MOESM1_ESM.docx]
